# HsdR Subunit of the Type I Restriction-Modification Enzyme EcoR124I: Biophysical Characterisation and Structural Modelling

**DOI:** 10.1016/j.jmb.2007.11.024

**Published:** 2008-02-15

**Authors:** Agnieszka Obarska-Kosinska, James E N Taylor, Philip Callow, Jerzy Orlowski, Janusz M. Bujnicki, G. Geoff Kneale

**Affiliations:** 1 Laboratory of Bioinformatics and Protein Engineering, International Institute of Molecular and Cell Biology, Trojdena 4, 02-109 Warsaw, Poland; 2 Biophysics Laboratories, Institute of Biomedical and Biomolecular Sciences, University of Portsmouth, PO1 2DT, UK; 3 EPSAM and ISTM Research Institutes, Keele University, Staffordshire ST5 5BG, UK; 4 ILL-EMBL Deuteration Laboratory, Partnership for Structural Biology, Institut Laue Langevin, 38042 Grenoble Cedex 9, Grenoble, France

**Keywords:** fold recognition, homology modelling, *de novo* modelling, DEAD box, SANS

## Abstract

Type I restriction-modification (RM) systems are large, multifunctional enzymes composed of three different subunits. HsdS and HsdM form a complex in which HsdS recognizes the target DNA sequence, and HsdM carries out methylation of adenosine residues. The HsdR subunit, when associated with the HsdS-HsdM complex, translocates DNA in an ATP-dependent process and cleaves unmethylated DNA at a distance of several thousand base-pairs from the recognition site. The molecular mechanism by which these enzymes translocate the DNA is not fully understood, in part because of the absence of crystal structures. To date, crystal structures have been determined for the individual HsdS and HsdM subunits and models have been built for the HsdM–HsdS complex with the DNA. However, no structure is available for the HsdR subunit. In this work, the gene coding for the HsdR subunit of EcoR124I was re-sequenced, which showed that there was an error in the published sequence. This changed the position of the stop codon and altered the last 17 amino acid residues of the protein sequence. An improved purification procedure was developed to enable HsdR to be purified efficiently for biophysical and structural analysis. Analytical ultracentrifugation shows that HsdR is monomeric in solution, and the frictional ratio of 1.21 indicates that the subunit is globular and fairly compact. Small angle neutron-scattering of the HsdR subunit indicates a radius of gyration of 3.4 nm and a maximum dimension of 10 nm. We constructed a model of the HsdR using protein fold-recognition and homology modelling to model individual domains, and small-angle neutron scattering data as restraints to combine them into a single molecule. The model reveals an ellipsoidal shape of the enzymatic core comprising the N-terminal and central domains, and suggests conformational heterogeneity of the C-terminal region implicated in binding of HsdR to the HsdS–HsdM complex.

## Introduction

Type I restriction-modification (RM) systems are large, oligomeric enzymes that can exhibit restriction endonuclease (REase) and/or DNA modification methyltransferase (MTase) activity.^1^, ^2^ They are composed of three different subunits encoded by three closely linked genes. The HsdS subunit is required for DNA recognition and specifies the target recognition sequence, HsdM binds *S*-adenosylmethionine (AdoMet) and carries out the methyltransfer reaction, while HsdR binds ATP and carries out the DNA cleavage. The mode of action of type I RM systems is quite sophisticated, as both HsdS and HsdM are required to form an active MTase, while a complex of all three subunits (including HsdR) forms a potent REase. The target DNA sequence is usually asymmetric, consisting of two half-sites 3–5 bp in length, separated by a non-specific spacer sequence 6–8 bp long.^3^ The activity of the complex as an MTase or REase is dependent upon the cofactors; AdoMet is essential both for methylation (as a methyl group donor) and for the cleavage (as a regulator), while the cleavage activity requires also ATP and Mg^2+^.^3^ The activity is determined by the methylation state of the target sequence; if the target sequence is methylated in one strand, the enzyme methylates the complementary strand efficiently. However, when the type I RM system encounters an unmodified target, it acts as an ATP-dependent molecular motor, pulling the DNA on either side toward itself. When this translocation is impeded by collision with another type I enzyme or by the topology of the DNA substrate, a double-strand break is introduced in the DNA at the collision point up to several thousands of base-pairs away from the target site.^4^, ^5^ The enzyme remains bound to the target site even after the cleavage reaction,^6^ and does not turn over as the nuclease, although ATP hydrolysis continues.^7^

The MTase core of a type I REase comprises two HsdM subunits and an HsdS subunit, with stoichiometry M_2_S_1_. The high-resolution crystal structure of a type I S subunit has been determined^8^, ^9^ and the structures of two type I M subunits have been deposited in the Protein Data Bank but have yet to be published. No crystal structure has been reported for the trimeric MTase; however, a low-resolution structure for the AhdI MTase has been determined by small angle neutron-scattering (SANS) that reveals the overall organisation of the subunits and domains of the enzyme.^10^

The enzymology and motor activity of the type I REase EcoR124I has been studied intensively. Two HsdR subunits bind sequentially to the 160 kDa MTase core to form the endonuclease (REase) with the stoichiometry R_2_M_2_S_2_^11^ However, R_1_M_2_S_1_ complexes can be formed and there is some uncertainty over the precise role of each of these complexes.^11^ There is a large difference in the binding affinities of the first and second HsdR subunits, with binding of the first HsdR subunit much stronger than binding of the second.^11^, ^12^ It has been reported that with one HsdR subunit bound, i.e. a stoichiometry of R_1_M_2_S_1_, no cleavage was observed, although the complex still functioned as an ATPase. Only with the addition of a second HsdR subunit does the REase cleave DNA.^11^ Nevertheless, the HsdR subunits of the REase work independently as molecular motors. The R_1_ complex has a translocation rate of 550(±30) bp s^−1^, while the rate of the R_2_ complex is twice this value.^13^

The HsdR subunit contains a number of domains responsible for DNA translocation, ATP hydrolysis and DNA cleavage.^14^, ^15^, ^16^ In particular, the HsdR subunit contains a DEAD-box helicase-like domain found in a diverse range of ATP-dependent enzymes,^17^ and implicated in DNA translocation in all type I R-M systems. Region X, which occurs N-terminally to the DEAD-box motif, shares similarities with the PD-(D/E)XK motif found in many type II REases and numerous other nucleases involved in DNA repair and recombination.^18^, ^19^, ^20^ The proline residue is absent from the type I consensus sequence. However, mutation studies on type II REases have suggested that it is not critical to activity.^21^

Although molecular structures of the HsdM and HsdS subunits of type I R-M systems are available, no structure has been published for a type I HsdR subunit. Here, we characterise the HsdR subunit of EcoR124I by dynamic light-scattering and analytical ultracentrifugation, and determine the shape of HsdR by SANS. Using protein-fold recognition and homology modelling techniques, we propose a detailed structural model for the HsdR subunit that is consistent with the SANS data.

## Results and Discussion

### Purification and characterisation of the HsdR subunit

**Figure 1:**
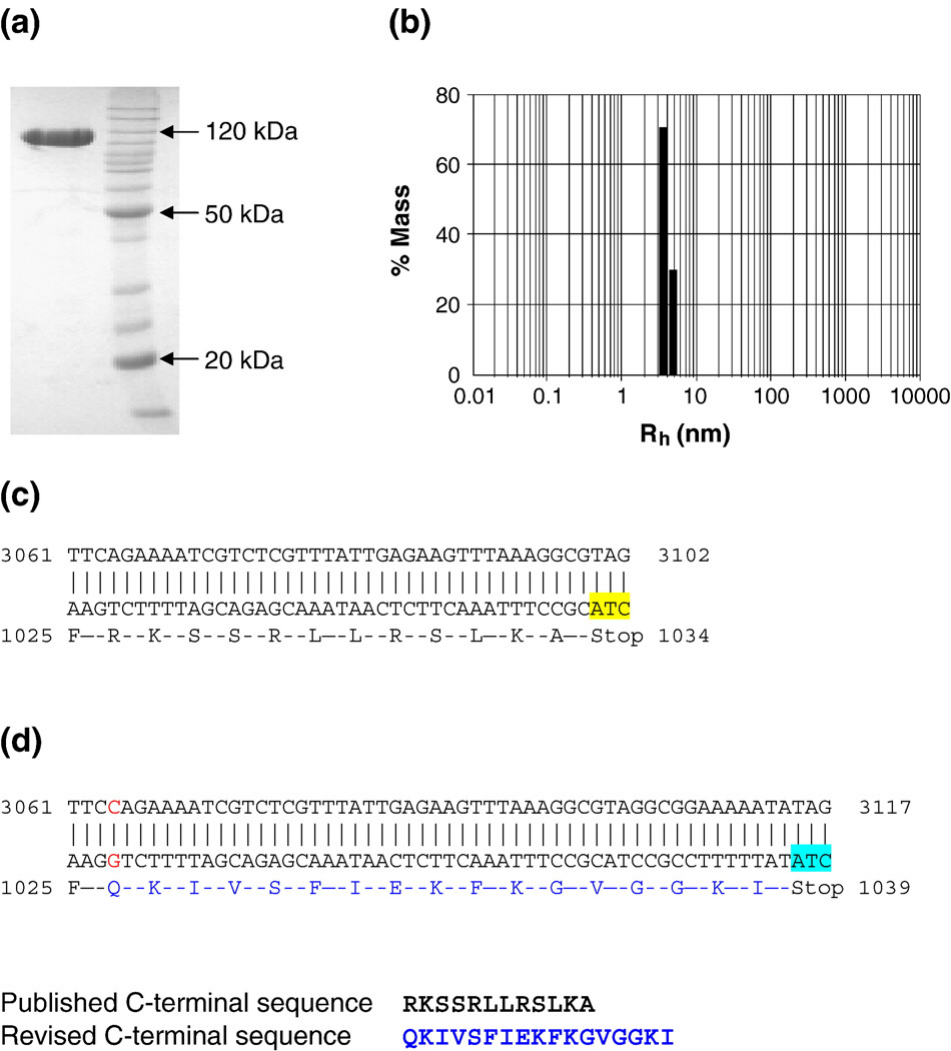
(a) SDS-PAGE. Purified HsdR was run on an SDS/12.5 % polyacrylamide gel and stained with Coomassie brilliant blue. The marker lane is labelled accordingly. (b) Dynamic light-scattering measurements of HsdR at 4.5 μM and 10 °C. (c) The published *hsd*R-gene sequence. (d) The revised *hsd*R-gene sequence. DNA sequencing revealed an additional nucleotide (red). The resulting frame-shift alters the translated amino acid sequence beyond this point and produces a new stop codon. The published stop codon is shown in yellow, and the new stop codon is shown in cyan. The revised C-terminal amino acid sequence of the R subunit is shown in blue.

As previous purification procedures for HsdR have been reported to result in proteolytic degradation and the loss of approximately 8 kDa of the protein,^22^ we developed a new purification procedure to minimise degradation before biophysical analysis. DNA-free cell lysates were prepared as described in Materials and Methods. The sample was applied to a desalting column that had been equilibrated in buffer A (10 mM Tris–HCl (pH 8.0), 100 mM NaCl, 1 mM Na_2_EDTA) and then applied to a heparin column equilibrated in the same buffer. The protein was eluted with a linear gradient of 0.1 M–0.6 M NaCl, desalted and applied to a Mono Q column and again eluted with a linear gradient of 0.1 M–0.6 M NaCl. This resulted in HsdR purified to greater than 98 % homogeneity (Figure 1(a)). No sign of degradation was observed by this new procedure.

Dynamic light-scattering was used to check the monodispersity of the purified protein (Figure 1(b)). HsdR was found to have a hydrodynamic radius of 3.8 nm, and an estimated molecular mass *M* of ∼94 kDa (Table 1). The *M* estimated by this technique depends on calibration standards and is not an accurate value; nevertheless, it is sufficient to rule out the presence of aggregation. The low level of polydispersity of the sample confirmed that it was suitable for further biophysical analysis at a concentration close to that used for subsequent experiments (4.5 μM).

**Table 1:** Dynamic light-scattering parameters of R.EcoR124I

Concn (μM)	*R*_h_ (nm)	Polydispersity		*M* (kDa)
		(nm)	(%)	
4.5	3.8	0.5	13	94

Mass spectroscopic analysis of the purified protein suggested that the size of the protein was ∼500 Da larger than expected from the original published sequence (Genbank X13145).^23^ To resolve this discrepancy, the *hsd*R-gene within the expression plasmid pBGSR124 was sequenced.^22^ The DNA sequence of the gene indicates an additional cytosine towards the 3′ end of the gene that was missing from the published sequence (Figure 1(c)). This frameshift has the effect of changing the sequence of 12 residues at the C-terminal end of the original protein sequence and extending the size of the translated protein sequence by a further five amino acid residues (Figure 1(d)). The revised sequence has been deposited with GenBank under accession number DQ249872. Further sequencing of the *hsd*R gene of the parental plasmid pCP1005, containing all three genes for the EcoR124I R-M system, showed that the sequence of the gene was identical with that in pBGSR124. This confirmed that no mutation had been introduced in the generation of the expression plasmid. On the basis of the revised amino acid sequence, the theoretical *M* of the HsdR subunit is 120,120 Da and the extinction coefficient is 98,225 M^−1^ cm^−1^.

### Analytical ultracentrifugation

**Figure 2:**
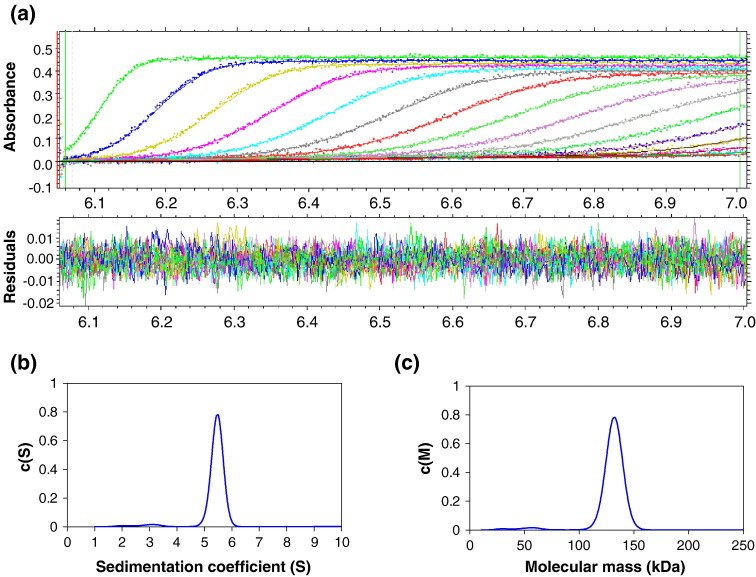
Sedimentation velocity of HsdR. (a) Data fitted from a run at 40,000 rpm (in an An50 Ti rotor) at a protein concentration of 4.5 μM, scanning at 280 nm and 10 °C, together with the corresponding residuals. For clarity, scans are shown for every fourth scan measured. (b) and (c) The associated *c*(S) and *c*(M) distribution plots.

Sedimentation velocity experiments were done with the purified EcoR124I HsdR subunit (Figure 2(a)). Data analysis was carried out using the program Sedfit,^24^ which describes the sedimentation data as a differential sedimentation coefficient distribution *c*(s). Using the estimated frictional ratio, the data can be transformed into a molar mass distribution *c*(M) to estimate *M* for each species. Sedimentation velocity of the R subunit at a concentration of 4.5 μM in buffer A revealed a single species with an experimental sedimentation coefficient (s*) of 5.5 and a corrected *s* value (*s*_20,w_) of 7.2 S (Figure 2(b)). The associated *c*(M) plot suggested an experimental molecular mass of approximately 130 kDa, in reasonable agreement with the revised theoretical *M* of 120 kDa (Figure 2(c)) and confirmed that the protein was monomeric (Table 2). There was no indication of a peak corresponding to dimers or higher aggregates. The frictional ratio determined by sedimentation velocity (*f*/*f*_0_ = 1.21) corresponds to a fairly compact globular protein. The Stokes radius from this analysis (4.0 nm) is in good agreement with the hydrodynamic radius estimated from dynamic light-scattering (3.8 nm). Similar measurements were performed in the presence of 100-fold excess of ATP and this gave an identical S value, suggesting that ATP does not cause any large-scale structural change when bound to HsdR.

**Table 2:** Sedimentation velocity parameters of R.EcoR124I

*M* (kDa)	s* (× 10^−13^ s)	*s*_20,w_ (× 10^−13^ s)	*R*_Stokes_^a^ (nm)	*f*/*f*_0_^a^
130	5.5	7.2	4.0	1.21

a Calculated from Sednterp using the theoretical *M*.

### Small angle neutron-scattering

**Figure 3:**
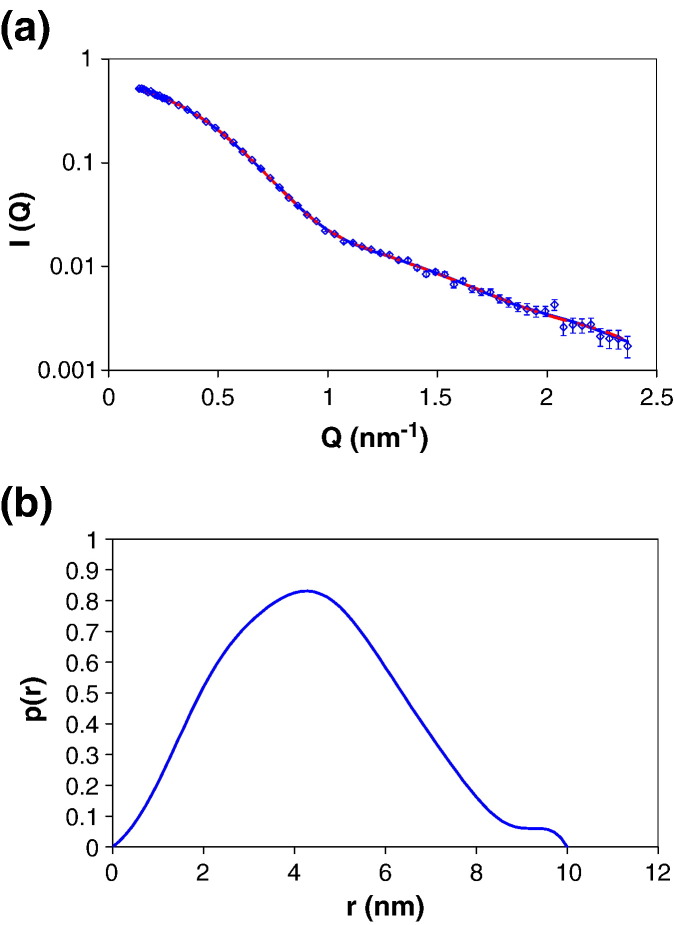
SANS data for HsdR in 100% H_2_O. (a) The points shown correspond to the experimental data; the lines represent the theoretical scattering curves calculated from the *p*(r) function (continuous blue line) and from the *ab initio* model. (broken red line). (b) Distance distribution function calculated from the experimental scattering curve.

Neutron-scattering data were collected for the purified HsdR at a concentration of 4 μM in 100% H_2_O buffer (Figure 3(a)). The scattering data can be transformed into a distance distribution function, *P*(r), which shows the distribution of all inter-atomic vectors in the molecule (Figure 3(b)). This allows us to determine the radius of gyration (*R*_g_ = 3.4 nm) and longest dimension (*D*_max_ = 10 nm) of the protein.

**Figure 4:**
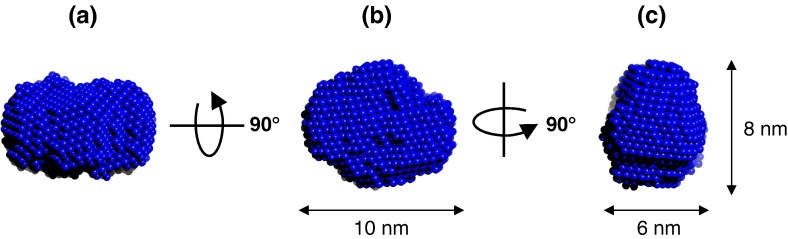
Low resolution dummy atom model for the HsdR subunit obtained from *ab initio* modelling of the SANS data. (a), (b) and (c) Three mutually perpendicular views of the structure.

*Ab initio* shape determination was then performed with the SANS data using the program DAMMIN.^25^ The modelling program was run 20 times and the resulting shapes averaged and filtered using the DAMAVER software suite to give the final structure.^26^ The shape determined for the HsdR approximates to that of an ellipsoidal structure with overall dimensions of 10 nm × 8 nm × 6 nm (Figure 4).

### Fold-recognition analysis of the EcoR124I HsdR subunit

In the absence of experimentally determined high-resolution structures, homology-based models may serve as convenient platforms for the investigation of sequence-structure-function relationships in proteins.^27^ In order to identify template structures for modelling of the HsdR subunit, we used the protein fold-recognition (FR) approach, which allows assessment of the compatibility of the target sequence with the available protein folds on the basis of the sequence similarity structural considerations (match of secondary structure elements, compatibility of residue-residue contacts, etc.). Since there is no homolog of known structure that can be used as a template to model the entire EcoR124I HsdR subunit, we searched for the templates for modelling its particular domains. We carried out a preliminary prediction of domain boundaries in EcoR124I HsdR subunit by searching the Conserved Domain Database (CDD) using RPS-BLAST^28^ and HHsearch.^29^ On the basis of the results of this analysis, we have split the HsdR sequence (1038 residues) into three overlapping segments, which were submitted independently to the GeneSilico meta-server for the three-dimensional fold identification.^30^ On the basis of the results of these preliminary FR analyses we finally divided EcoR124I HsdR into the following domains: N-terminal nuclease domain (residues 1–249), DNA translocase module (residues 250–728), composed of two RecA-like NTPase domains (residues 250–464 and 473–728) and C-terminal domain (residues 729–1038), and we have re-run FR analyses for these sequence fragments.

**Figure 5:**
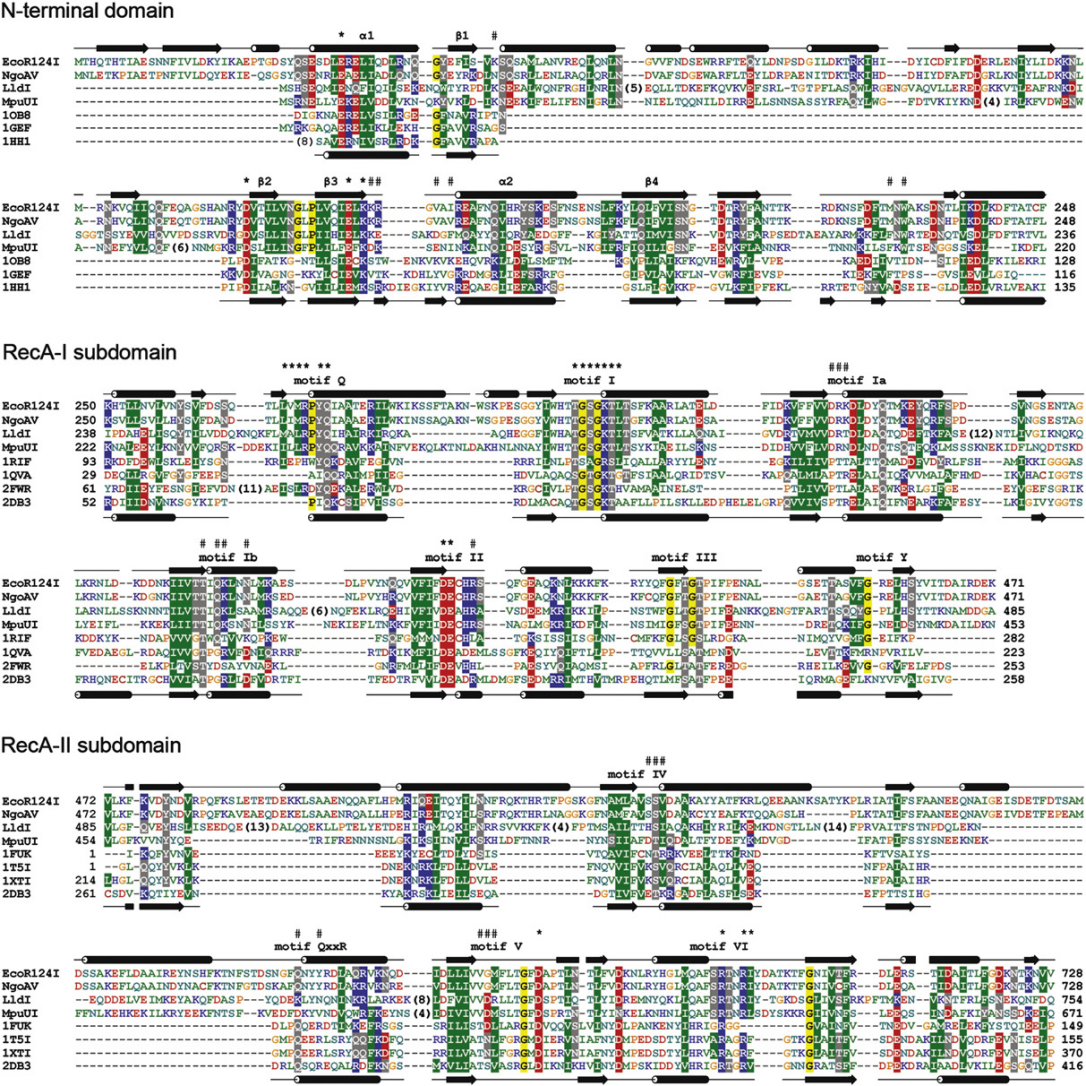
Sequence alignment of the EcoR124I HsdR and its homologs and the templates used for modelling the NTD, RecA-I and RecA-II subdomains. Similar amino acids are coloured according to the physico-chemical properties of their side-chains: negatively charged, red, positively charged, blue, polar, magenta, hydrophobic, green. Sequences are named according to nomenclature from REBASE or PDB codes. Numbers in parentheses indicate how many amino acid residues have been omitted for the sake of clarity. Amino acid residues of the NTD that are predicted to form a catalytic site and residues of the central domain that are predicted to be involved in ATP binding are indicated above the alignment by an asterisk (*). Putative DNA-binding residues are indicated by a hash mark (#). The secondary structure of the EcoR124I domains derived from the model using the DSSP program is shown above the EcoR124I sequence. Secondary structure of the templates: 1hh1 and 2db3 shown below the sequences. β-Strands are shown as arrows and helices are shown as cylinders.

For the N-terminal domain (NTD), which is known to possess a nuclease active site, the FR servers were expected to identify structures of nucleases from the PD-(D/E)XK superfamily as the preferred templates.^19^ However, most servers failed to report significant similarity to any protein of known structure (data not shown). Nevertheless, analysis of sequence conservation indicated that the NTD of EcoR124I HsdR harbours a conserved pattern of residues E-Xn-D-X13-E-X-K (Figure 5) resembling the canonical PD-(D/E)XK nuclease motif ((E)-Xn-(P)D-Xn-(D/E)-X-K). Moreover, a prediction of secondary structure indicated that the conserved residues are associated with an α-β-β-β-α-β pattern of secondary structures typical for nucleases from the PD-(D/E)XK superfamily.^19^, ^20^ However, according to predictions of secondary structure, the PD-(D/E)XK domain of the EcoR124I HsdR contains a long insertion (residues 58–146) between the first (β1) and the second β-strand (β2) of PD-(D/E)XK fold and additional two β-strands at the N terminus (Figure 5). Therefore, we carried out additional FR analyses of the NTD using a sequence without that insertion and without the 30 residues extension at the N terminus. In this case, all servers from the GeneSilico MetaServer reported matches to structures of PD-(D/E)XK nucleases as the best modelling templates. The structure of a Holliday junction resolvase (Hjc) from *Sulfolobus solfataricus* (PDB code 1hh1)^31^ was identified as the best template by most primary FR servers, as well as by the PCONS consensus server (score 0.6811). The structure of a related Hjc resolvase (PDB code 1ob8)^32^ was proposed by many servers and selected by PCONS as the second best template (score 0.6035). The third best template reported by PCONS was the structure of resolvase Hjc from *Pyrococcus furiosus* (PDB code 1gef)^33^ (PCONS score 0.5918). Thus, these three structures (1hh1, 1ob8 and 1gef) have been used as templates for modelling the nuclease domain of EcoR124I HsdR using the FRankenstein's Monster approach.

The central region, which was predicted to function as the DNA translocase,^17^ was unambiguously predicted to exhibit the DEAD-box helicase-like fold with the tandem repeat of two RecA-like (AAA) subdomains. All servers reported statistically significant matches to known structures of DEAD-box helicases and related enzymes, and indicated that the core RecA subdomains in HsdR span residues 250–464 and 473–728, respectively. To obtain more accurate target-template alignments, we carried out FR analysis for each of NTPase domain independently, using sequences without fragments corresponding to apparent insertions. The structure of UvsW helicase from bacteriophage T4 (PDB code 1rif)^34^ was reported by PCONS (score 3.0563) as the best template for modelling the RecA-I subdomain, while yeast EIF 4A (PDB code 1qva)^35^ was reported as the second best template (score 2.9238). The XPB helicase from *Archaeoglobus fulgidis* (PDB code 2fwr) was proposed as the third best template. The yeast EIF4A (PDB code 1fuk) was reported as the best template for modelling the RecA-II subdomain (PCONS score 2.8148), followed by human UAP56 (PDB codes 1xti and 1t5i)^36^, ^37^ (PCONS score 2.775). Therefore, the above-mentioned structures were selected as the templates for modelling the RecA-like subdomains (three templates for each subdomain). The relative orientation of two subdomains in HsdR and conformation of the regions at the domain interface was predicted on the basis of the structure of the RNA-unwinding DEAD-box protein from Drosophila Vasa (PDB code 2db3),^38^ which was reported as the top template (PCONS score 3.4068), when the entire central domain (with both subdomains and a linker sequence) was submitted for FR analysis.

The C-terminal domain (CTD) of HsdR (residues 729–1038) is believed to be important for binding HsdR to the HsdS-HsdM complex.^39^ In our FR analyses, it showed no significant similarity to any proteins with known tertiary structure. However, secondary structure prediction indicated that this region is rich in helices, and exhibits some tendency for intrinsic disorder (according to DISPROT^40^) and shows a potential to form two to four coiled coils. The consensus of coiled coil predictions suggested the presence of two long helices in regions corresponding to residues 838–869 and 956–977, forming a coiled-coil structure. Interestingly, the first structural template proposed for the CTD of HsdR by the GeneSilico MetaServer (C-terminal domain of turkey PLC-beta; (PDB code 1jad) revealed an antiparallel coiled-coil fold and the proposed alignment that agreed with the *de novo* coiled-coil prediction. Thus, we generated an additional model for a fragment of the CTD (residues 838–869 and 956–977), represented as a coiled coil.

### Modelling of domains of the EcoR124I HsdR subunit and evaluation of the models

Target-template alignments proposed by FR servers for the nuclease core and central domains of HsdR domains exhibited slight differences. Therefore, comparative models of these domains were built using the FRankenstein's Monster approach that attempts to optimize the sequence-structure compatibility (as assessed by MetaMQAP), while retaining the consensus regions of the alignment (see Materials and Methods).

The final sequence alignment of the NTD of EcoR124I HsdR and its homologs and the templates used for modelling the PD-(D/E)XK nuclease core is shown in Figure 5. Regions of the EcoR124I HsdR comprising residues 1–30 and 58–146 have no counterpart in any template structure and were omitted from the template-based modelling. The N-terminal extension at the N terminus was added *de novo* with ROSETTA (starting from an extended loop protruding out of the comparative model of the domain core). We also attempted *de novo* modelling of residues 58–146. However, this polypeptide fragment behaved essentially as an independently folded domain (data not shown); therefore, we decided to fold it separately, without the rest of the protein (to reduce the time of calculations and thereby improve sampling of the conformational space). The most common conformation obtained in the course of the folding of the insertion alone was actually very similar to those obtained together with the rest of the domain, supporting our prediction of its “independent” structure. The final model of the NTD was obtained by docking of the insertion into the PD-(D/E)XK core (see Materials and Methods).

To build a low-resolution model of the EcoR124I NTD in complex with DNA we searched for DNA-bound structures of PD-(D/E)XK nucleases structurally most similar to the 1gef template. Six structures (PDB codes 1fok, 1cw0, 1iaw, 3pvi, 1dmu and 1fiu) identified by DALI^41^ were superimposed onto the NTD model. For each structure, we checked if its DNA part could be transferred to form a EcoR124I NTD–DNA complex without major steric clash with the protein. We found that DNA from the type II restriction enzyme NgoMIV from *Neisseria gonorrhoeae* (PDB code 1fiu) solved in the complex with DNA and magnesium ions showed best shape complementarity with the NTD model.^42^ Although NgoMIV contains a non-canonical PD-SXK motif in which the conserved (D/E) carboxylate has been replaced by Ser, an alternative carboxylate group coming from non-homologous structural element is present and the arrangement of the conserved catalytic residues is the same as in typical PD-(D/E)XK nucleases. Therefore, we used the NgoMIV structure as the template for modelling the EcoR124I NTD–DNA complex, into which the coordinates of magnesium ions and the DNA molecule (chains E, I, F, J) were copied from the NgoMIV structure. Since DNA in the NgoMIV structure is cleaved, we ligated broken phosphodiester bonds to form a contiguous DNA structure. We emphasize here that the resulting protein–DNA model is of low resolution and serves only to illustrate a possible orientation of the nuclease domain and its substrate DNA.

A comparative model of the DNA translocase module was constructed based on the basis of the iterative optimization of the sequence-structure fit with the FRankenstein's Monster method (Figure 5). The insertions that have no counterpart in any of the template were modelled using ROSETTA. Finally, the ATP molecule and magnesium ion were added by copying the coordinates of the ATP and magnesium ion from the 2db3 template structure.

Theoretical models of protein structure must be evaluated carefully before they are used for functional interpretation. Unlike crystallographic structures, they are not based on experimental data from which the electron density is inferred, but on identification of evolutionary relationships, assignment of correspondence between amino acid residues, and only to a limited extent on sampling of the conformational space. Template-based modelling methods as well as fragment-based *de novo* folding methods such as ROSETTA copy bond lengths and angles from previously determined protein structures to generate protein-like conformations; therefore the assessment of e.g. the Ramachandran plot in theoretical models has no practical value (it cannot be used as a control variable similar to the common use in crystallography). Calculations of physical energy are also useless for the assessment of theoretical models such as those reported in this work, as it has been shown that even very limited deviation from the native conformation (e.g. RMSD ∼ 0.2 nm) causes the energy to increase to the level indistinguishable from that of completely misfolded models.^43^ Thus far, the only reliable methods for quality assessment of protein models (the so-called MQAPs) are based on statistical analyses of previously solved structures, which describe quantitatively the “protein-likeness” of intramolecular contacts and various generic parameters, such as packing, charge complementarity, burial of hydrophobic side-chains etc.^44^

**Figure 6:**
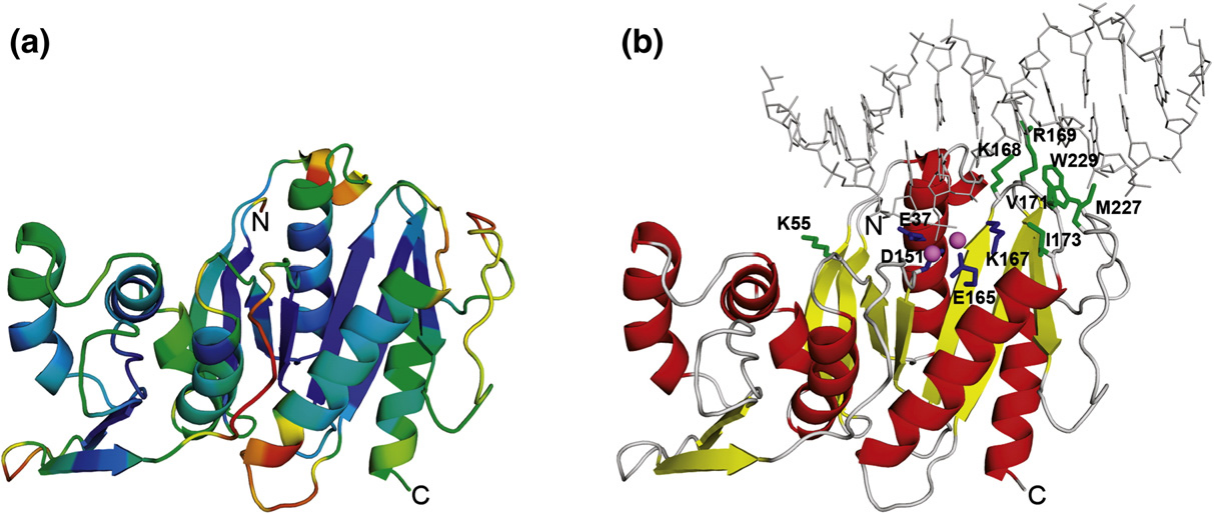
Model of the NTD of EcoR124I HsdR. (a) Model coloured according to MetaMQAP evaluation (well-scored regions are coloured blue, poorly scored regions are coloured red). (b) Residues predicted to be functionally important. The residues predicted to form the active site are shown in blue. The putative DNA-binding residues are shown in green. Two magnesium ions are shown as pink spheres and the DNA molecule is shown as grey lines. The protein chain is coloured according to secondary structure (red, helices; yellow, β-strands; grey, loops).

**Figure 7:**
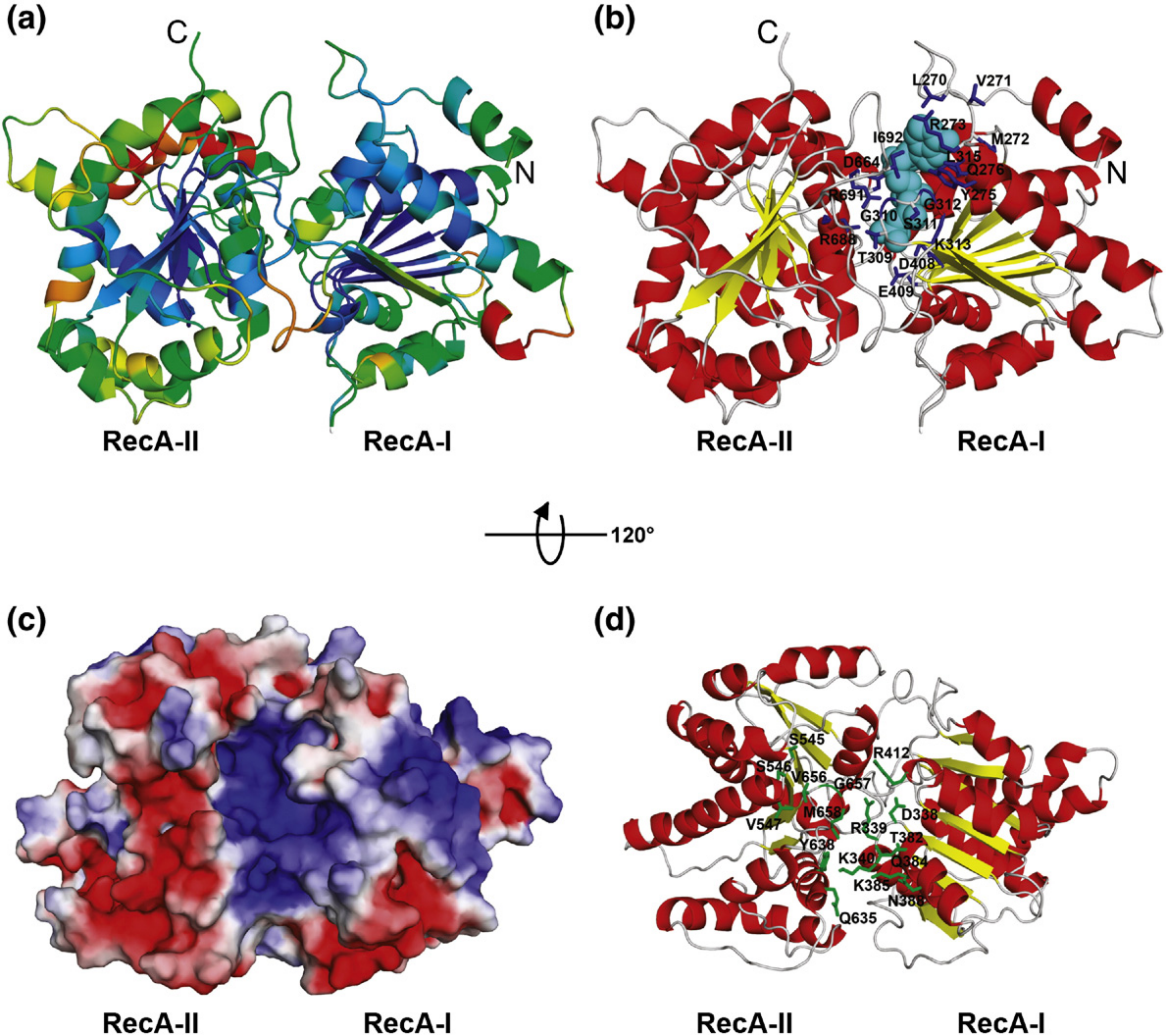
Model of the central domain of EcoR124I HsdR. (a) Model coloured according to MetaMQAP evaluation (well-scored regions are coloured blue, poorly scored regions are coloured red). (b) Active site of the central domain: ATP moiety (cyan) and magnesium ion (pink) coordinates copied from 2db3 structure. The residues predicted to be involved in ATP-binding (shown in blue) are located in a cleft between two NTPase domains. The model is coloured according to secondary structure (red, helices; yellow, β-strands; grey, loops). (c) Electrostatic potential mapped onto the molecular surface of the central domain (positively and negatively charged regions are coloured in blue and red, respectively). (d) Putative DNA-binding residues (coloured green).

MetaMQAP recently developed in our group uses parameters computed by several different MQAP methods (Materials and Methods) to predict the absolute deviation of C^α^ atoms for each residue in the model from its counterpart in a native structure, without any knowledge of the actual native structure. MetaMQAP was used within the FRankenstein's Monster protocol for evaluation of models and selection of the best fragments. According to this method, the predicted RMS deviation between the final models of NTD and the central domain from the (unknown) native structure of EcoR124I is 0.45 nm. This predicted accuracy is moderate, significantly lower than those of crystal structures, but sufficient for making functional inferences. Figure 6, Figure 7 show models of the NTD and central domain of EcoR124I HsdR subunit coloured according to the predicted RMS deviation,^45^ which indicates regions of higher and lower levels of accuracy. Importantly, regions predicted to be functionally important (e.g. catalytic and ligand-binding residues, and structures in the protein core) exhibit relatively high levels of accuracy, while regions predicted to be less accurate are located in functionally less relevant parts of the protein structure.

In order to provide independent assessment of the accuracy of our predictions, we submitted the models of EcoR124I HsdR domains to the ProQ server.^46^ According to ProQ, the model of the entire NTD was evaluated as a “fairly good model” (predicted LGscore 2.088; without insertions modelled with ROSETTA, the predicted LGscore is 2.445, close to a “very good model”). The model of the DNA translocase domain obtained a predicted LGscore of 2.218 (which indicates a fairly good model), but without insertions the score increased to 2.794 (which indicates a very good model).

In summary, the scores for the NTD indicate that the three-dimensional fold of the PD-(D/E)XK core has been predicted correctly and the mutual position of most residues is reasonable, enabling predictions of DNA-binding loops etc. Nonetheless, with the exception of the PD-(D/E)XK active site, the conformations of most individual side-chains in the PD-(D/E)XK domain are not modelled reliably, and in this respect the current model should not be over-interpreted. The insertion (residues 58–146) modelled with ROSETTA gave much worse scores (e.g. ProQ predicted LGscore 0.911) and must be regarded as uncertain. The structural cores of both NTPase domains and the interface between both domains can be regarded as confident (within the estimated accuracy for individual residues), which enables prediction of amino acids involved in ATP and DNA binding. Only the insertions modelled with ROSETTA and some peripheral helices have to be regarded as uncertain. Thus, according to MetaMQAP and ProQ, homology-modelled parts of our model are significantly more reliable than those modelled *de novo*, in agreement with common sense and results of CASP.

### Functional interpretation of models for individual domains

In the proposed model of the NTD, the spatial configuration of the catalytic residues is typical for an active site architecture conserved among PD-(D/E)XK nucleases. In agreement with the alignment between HsdR sequences of EcoR124I and EcoKI, for which the active site has been verified experimentally,^39^ we find that the active site of the EcoR124I nuclease domain comprises residues D151, E165 and K167. We have identified another putative catalytic residue, E37 in the first helix (α1) of the PD-(D/E)XK fold, which has not been identified previously (Figure 5). On the basis of homology of this domain to type II restriction enzymes, we have used a structure of the NgoMIV-DNA-Mg^2+^ complex to model the corresponding interactions in HsdR. The homology-based prediction of DNA-binding residues agrees very well with *de novo* (structure-based) prediction of DNA-binding sites done with the PreDs server.^47^ Thus, we predict that the following residues of EcoR124I HsdR are likely to be important for binding of the DNA by the nuclease domain K55, K168, R169, V171, I173, M227, W229. The charged residues K55, K168 and R169 may be involved in interactions with DNA bases or backbone but the resolution of the model is too low to predict the interactions in atomic detail. The hydrophobic residues V171, I173, M227 and W229 may be involved in van der Waals interactions with bases in the major groove (Figure 6(b)). Notably, the structural superposition of the NgoMIV and EcoR124I HsdR reveals that these hydrophobic residues superpose with charged and polar residues responsible for specific DNA sequence recognition in the NgoMIV-DNA complex.^42^ This difference is in good agreement with the fact that NgoMIV and EcoR124I cleave within specific and non-specific sequences, respectively.

Our model of the DNA translocase module represents the HsdR sequence threaded along the generic SFII helicase scaffold, and can be used to illustrate generic features common to SFII enzymes, such as ATP binding and nucleic acid binding. Unfortunately, the resolution of our model is too limited to provide mechanistic explanation for its double-stranded DNA translocase activity, as opposed to helicases or for specificity for DNA, as opposed to RNA. On the basis of the homology of the central region of EcoR124I HsdR to known ATP-dependent SFII helicase structures, the spatial configuration of its ATP-binding site can be inferred. We predict that the following residues of EcoR124I HsdR are involved in ATP binding: 270-LVMR-273, Y275 Q276 (motif Q), 309-TGSGKTL-315 (motif I) D408, E409 (motif II), D664 (motif V), R688, R691 and I692 (motifVI) (Figure 7(b)). The distribution of the electrostatic potential on the protein surface (Figure 7(c)) suggests that the DNA-binding site is localized in the cleft between the two RecA-like domains (Figure 7(d)), similar to the RNA-binding site in the template structure 2db3. This prediction is supported also by the results of the PreDs server for prediction of DNA-binding sites (data not shown). On the basis of our model we propose that the following residues are likely to be involved in DNA binding by the central translocase module: 338-DRK-340 (motif Ia), T382, Q384, K385, N388 (motif Ib), R412 (motif II), 545-SSV-547 (motif IV), Q635, Y638 (motif QxxR), 656-VGM-658 (motif V) (Figure 7(d)). According to our model, motif Y is not involved in interactions with DNA as proposed by McCelland *et al*.^16^ Instead, it appears to have mostly a structural role.

### Rigid body modelling coupled with addition of missing fragments

**Figure 8:**
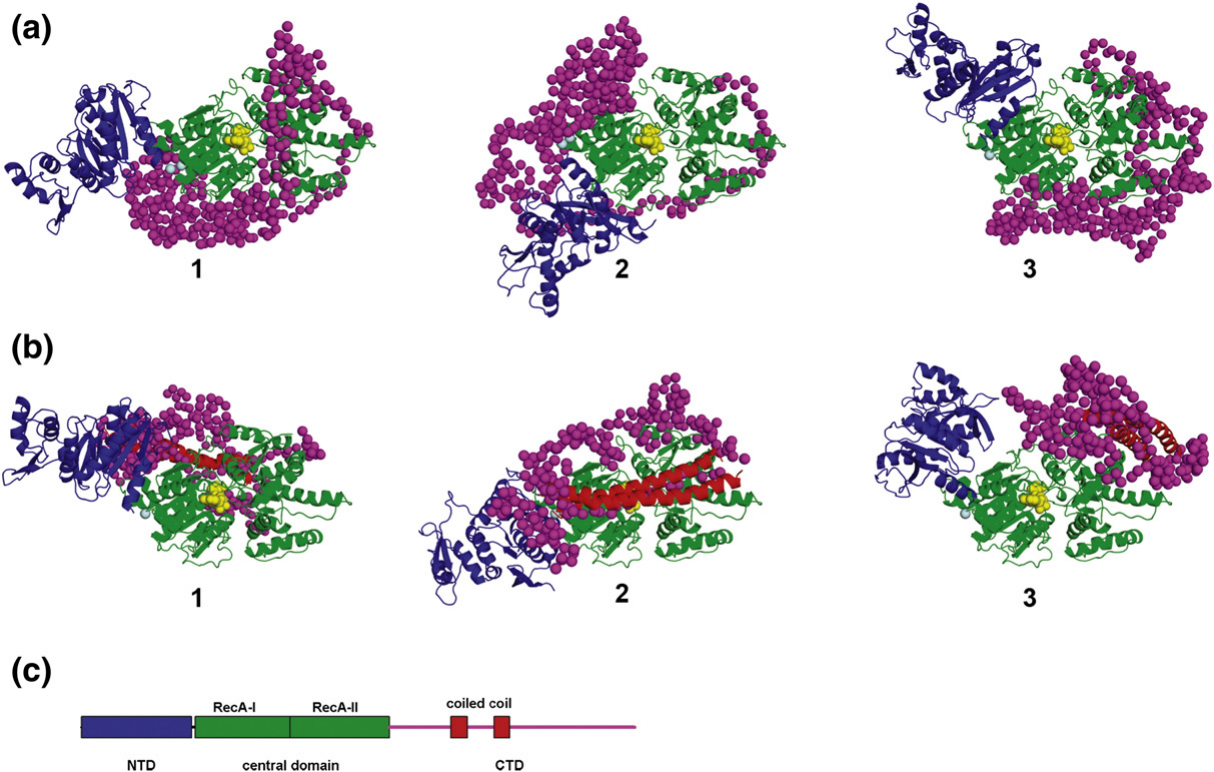
Determination of the structure of the entire HsdR subunit against SANS data. NTD (blue), central domain (green) and coiled-coil regions (red) are shown as cartoons, the linker between NTD and central domain (cyan) and CTD (magenta) are shown in a low-resolution dummy atom model. The ATP molecule is shown in yellow. (a) Three representative reconstructions made using homology models of NTD and central domains with (a1) χ = 1.51 (a2) χ = 1.54 (a3) χ = 1.59. (b) Three representative reconstructions made using homology models of NTD, central domain and coiled-coil regions of CTD (b1) χ = 1.41 (b2) χ = 1.46 (b3) χ = 1.49. (c) Domain architecture of the EcoR124I HsdR subunit.

Having the full-atom models of the NTD and central domain as well as the fragment of CTD of HsdR, we attempted to determine the overall structure of the HsdR subunit using the information from the SANS experiment. We used a combined rigid-body and *ab initio* modelling approach as implemented in program BUNCH.^48^ The program finds optimal positions and orientations of modelled fragments simultaneously by moving them as rigid bodies, and models the rest of the structure *de novo*. We performed multiple reconstructions using only the models of the NTD and central domain, and we obtained many possible models (Figure 8(a)), all yielding good fits to the scattering curve (χ values between 1.43 and 1.68). Using additionally a model of the coiled-coil fragment from the CTD, we also obtained many possible reconstructions (Figure 8(b)), with χ values between 1.41 and 1.69.

The variability of domain orientations in the reconstructed models suggests that the HsdR subunit may be conformationally flexible (i.e. that the orientation of domains is not fixed, and/or the CTD may be at least partially unstructured in the absence of HsdM-HsdS subunits) or that our SANS data are of insufficient resolution to identify a single family of conformations. The first explanation is supported by the following observations. (I) Bioinformatics methods predict that the CTD contains regions of intrinsic disorder, a feature that is not uncommon among polypeptide regions involved in protein–protein interactions. In a related enzyme, EcoKI, the C terminus is susceptible to degradation by proteases,^39^ supporting this prediction. (II) The nuclease complex must undergo conformational changes in the course of EcoR124I activity. It is very likely that the NTD nuclease domain is kept away from the DNA to avoid cleavage until it is recruited to cut the substrate under particular conditions. We predict that HsdR assumes a more defined structure when in complex with the HsdM-HsdS core. However, it must be emphasized that even in such a context it is likely to exhibit multiple conformations, depending on the presence of the ligands (ATP, DNA), and the particular functional state (DNA translocation, cleavage etc.).

## Conclusions

We have obtained biophysical data from analytical ultracentrifugation and SANS experiments, which indicate that the HsdR subunit is monomeric, globular and fairly compact, with a radius of gyration (*R*_g_) of 3.4 nm, a maximum dimension (*D*_max_) of 10 nm and a frictional ratio of 1.21.

Using a combination of fold recognition, homology modelling, and *de novo* protein folding methods, we constructed three-dimensional models of the NTD and central domain of the EcoR124I HsdR structure, and we predicted that the CTD contains a coiled-coil element, presumably involved in protein–protein interactions. The template-based models of the PD-(D/E)XK nuclease and the central DNA-translocase module comprising two RecA-like domains should be regarded as confident with respect to the tertiary fold and the localization of ligand-binding/catalytic residues. The uncertain regions have been delineated and will be refined or corrected as more experimental data become available. In the absence of a high-resolution crystal structure of the HsdR subunit, our models of its NTD and central domain will serve as a convenient platform to study sequence-structure-function relationships in this protein and in connection with our previous model of the HsdS-HsdM2 complex,^49^ will facilitate the development of an experimentally validated model of the entire type I RM enzyme complex.

We used the predicted structures of the HsdR domains, together with constraints from the SANS data, to calculate possible models of the entire HsdR subunit, which revealed considerable conformational heterogeneity within the spatial constraints provided by current experiments. A dynamic structure of HsdR agrees with the predicted intrinsic disorder of its C-terminal region and relatively flexible connection between individual domains. These models may serve as a useful platform for the design of experiments aiming at the determination of points of possible interactions between the individual domains within the HsdR subunit, as well as between HsdR and the MTase core; for instance, using residue-specific cross-linking,^50^ labelling and fluorescence resonance energy transfer (FRET) or electron paramagnetic resonance (EPR) experiments,^51^ or SANS experiments on the multisubunit REase using selective subunit deuteration techniques.^10^

## Materials and Methods

### Expression and purification

Competent cells of *Escherichia coli* strain BL21(DE3) were transformed with the plasmid pBGSR124 encoding the HsdR subunit.^22^ The bacteria were then grown at 30 °C on Enfors minimal medium using an Infors fermentation system to an absorbance at 600 nm of ∼15. Cell pellets were resuspended in 25 ml of lysis buffer (50 mM Tris–HCl (pH 8.0), 100 mM NaCl, 25 % (w/v) sucrose, 5 mM EDTA and 3 mM DTT) per 1 l of culture, at 4 °C. The cells were lysed using a Vibracell™ VCX 500 high-intensity ultrasonic processor (Jencons-PLS) with the CV 33, 0.5 in probe. The amplitude was set to 40%, with a maximum temperature of 10 °C. The cells were lysed for a total of 5 min with pulses of 9 s on and 9 s off.

DNA was removed following the removal of insoluble macromolecules and cell debris. Protamine sulphate (Sigma) and NaCl were added to the lysate to final concentrations of 20 mg/ml and 500 mM, respectively. The solution was mixed at 4 °C for 30 min before precipitated nucleic acids were removed from the sample by centrifugation (12,000***g*** at 4 °C for 20 min). The sample was desalted using a HiPrep™ 26/10 desalting column (GE Healthcare) that had been equilibrated in buffer A (10 mM Tris–HCl (pH 8.0), 100 mM NaCl, 1 mM Na_2_EDTA) and then applied to a HiPrep™ 16/10 heparin FF column, equilibrated in buffer A. After injection of the sample containing the R protein, the column was washed with buffer A and the protein was eluted with a linear gradient of NaCl (0.1 to 2.0 M) at a flow rate of 1 mL/min over 20 column volumes. The eluted sample was desalted using a HiPrep™ 26/10 desalting column (GE Healthcare) equilibrated in buffer A and then applied to a Mono Q HR 5/5 (GE Healthcare) column equilibrated in the same buffer. Following injection, the column was washed with buffer A, before elution with a linear 0.1M–0.6 M NaCl gradient over 20 column volumes.

### Dynamic light-scattering (DLS)

DLS was performed with purified HsdR at 4.5 mM, at 10 °C in buffer A, using a Protein Solutions DynaPro MSTC800 instrument. The results from 30 measurements were averaged, and values for the hydrodynamic radius, *R*_h_, and polydispersity, were obtained. The experimental molecular mass *M* was estimated using a volume shape hydration model based on a range of proteins of 24–100 kDa with an average partial specific volume of 0.726 and frictional ratio of 1.257.

### Analytical ultracentrifugation

Sedimentation velocity was carried out using 400 ml of HsdR at 4.5 mM and 425 ml of buffer A in a double-sector cell of 12 mm optical path-length. The cells were loaded into an AN50-Ti analytical rotor, and left to equilibrate to 10 °C. The rotor was accelerated to 40,000 rpm and readings of absorbance *versus* radial distance were taken every 12 min at 280 nm. The raw data were analysed using the program Sedfit,^24^ using radial data within the range 6.06 cm–7.00 cm for the first 52 scans. Partial specific volumes and buffer densities were calculated using the program Sednterp and corrected for temperature.^52^ The experimental sedimentation coefficients obtained from the *c*(S) distribution plot (Sedfit‡), were finally corrected for temperature and solvent using Sednterp, so that a value for *s*_20,w_ could be obtained.

### Small angle neutron-scattering

Scattering curves were collected using the D22 diffractometer at the ILL, Grenoble, with two detector distances covering a *Q* range of 0.1 nm^−1^–3.0 nm^−1^. Measurements were made at a protein concentration of 4 μM in buffer A at 10 °C. Data reduction was performed using the GRAS_ans_P software§.

Further analysis of the SANS data was performed using the ATSAS software package developed by Svergun *et al*.∥ Distance distribution functions, *P*(r), were calculated using GNOM.^53^ The *R*_g_ values derived from the *P*(r) function were closely comparable with those derived from the Guinier plot.

### *Ab initio* structural modelling based on SANS data

*Ab initio* shape determination was performed using DAMMIN,^25^ which uses simulated annealing to calculate single-phase dummy atom models. A sphere was defined with a diameter of 10 nm (corresponding to the calculated *D*_max_ from the *P*(r) function) composed of 1926 dummy atoms each with radius of 0.36 nm. No symmetry was imposed and the simulated annealing procedure was run with a schedule factor of 0.9 to model the data. Models with *R*_f_, Looseness and Disconnectivity values of greater than 0.01, 0.1 and 0.0, respectively, were discarded.

The data were modelled 20 times and the resulting shapes were aligned, averaged and filtered using the DAMAVER package of programs.^26^ The cut-off volume for the resulting shape after filtering was varied until the *R*_g_ of the shape calculated with the program CRYSON corresponded to that determined experimentally.^54^

### Protein structure prediction

Searches of homologs of the HsdR subunit of the EcoR124I were carried out using BLAST at the databases: REBASE^55^ and the non-redundant (nr) database at the National Center for Biotechnology Information.^56^ A multiple sequence alignment was generated using MUSCLE,^57^ and optimized manually on the basis of the results of structural analyses. Prediction of coiled coils from protein sequence was done using COILS and PCOILS servers,^58^ and MARCOIL.^59^

Prediction of domain boundaries in the EcoR124I HsdR subunit was carried out by searching the CDD database of known domains using RPS-BLAST^28^ and Hhsearch.^29^ Sequences corresponding to individual domains were submitted independently to the GeneSilico MetaServer,^30^ which is a gateway for a variety of methods for making structure predictions and analyzing their results, in particular for a set of FR methods that align the target sequence with potential modelling templates. Target-template alignments reported by FR methods were compared, evaluated, and ranked by the PCONS server^60^ to identify the preferred modelling template and the consensus alignment. They have been used also to carry out comparative modelling of the HsdR structure using the FRankenstein's Monster approach, which comprises cycles of local realignments in uncertain regions, building of alternative models and their evaluation, realignment in poorly scored regions and merging of the best-scoring fragments. This method was found to be one of the most accurate approaches for comparative modelling and FR in the rankings of CASP5 and CASP6.^61^, ^62^ Examples of structures of restriction enzymes successfully predicted with this methodology (i.e. models confirmed by crystal structures) include R.SfiI^63^ and R.MvaI,^64^ as well as other nucleases from the PD-(D/E)XK superfamily.^65^, ^66^

Insertions, for which no reliable modelling template was found, were modelled *de novo* using ROSETTA,^67^ which attempts to generate native-like global conformations from three and nine residue backbone fragments of experimentally solved protein structures. Fragment selection is based on profile–profile comparison of sequence and secondary structure between the target sequence and the database of fragments derived from the PDB database. ROSETTA is one of the best methods for *de novo* modelling and is capable of adding structurally variable regions (insertions) to the core built with comparative modelling methods.^68^ We carried out the fragment assembly with default parameters and a medium level of side-chain rotamers optimization. The most common conformation of each insertion in the set of obtained decoys was chosen by clustering decoys on the basis of structural similarity of the given insertion. The largest insertion (59–149 residues) in the PD-D/EXK domain was modelled independently of the rest of the protein, and subsequently inserted into the core domain using the docking method HADDOCK,^69^ with distance restraints that ensured the possibility of joining peptide ends to reproduce a continuous polypeptide backbone.

For the evaluation of models we used two methods: PROQ^44^ and MetaMQAP recently developed by our group¶. They predict the overall quality of the model and deviation of individual residues in the model from their counterparts in the native structure.

### Rigid body modelling of predicted domains coupled with addition of missing fragments

Assuming that the predicted structures of individual domains of HsdR were approximately correct, we attempted to reconstruct their mutual orientation in HsdR using the BUNCH program from the ATSAS package.^48^ We performed multiple reconstructions with default parameters in two versions: (1) using only the models of the NTD (residues 1–249; nuclease) and central domain (residues 250–728; translocase) and the remaining residues as undefined; and (2) using the above-mentioned domains and a model of the coiled-coil fragment of the CTD (residues 827–868 and 956–989). The neutron-scattering amplitudes from the domains or domains fragments were computed using CRYSON.^54^

All visualisations of PDB files were performed using PyMol^a^.

## Figures and Tables

**Supplementary:**
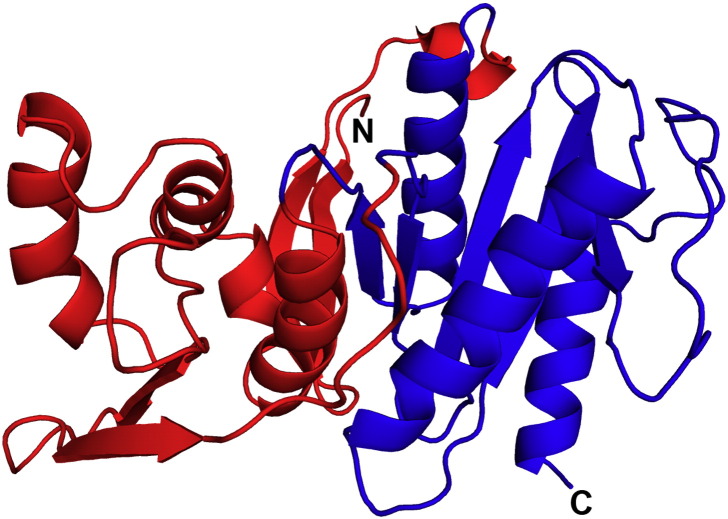


**Supplementary:**
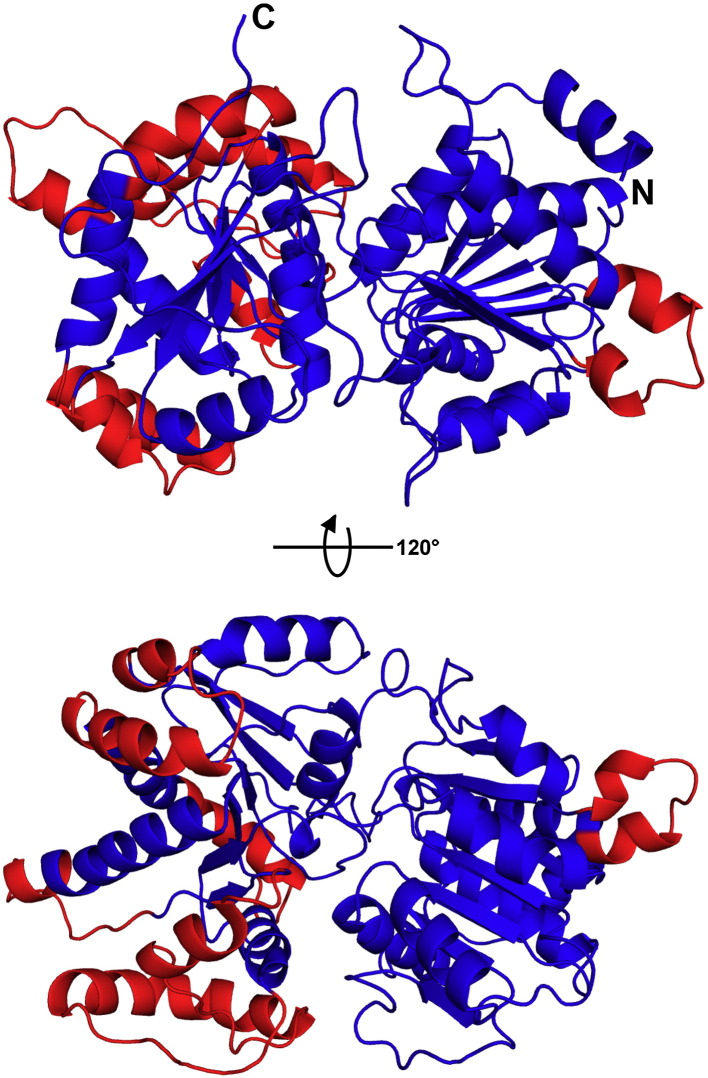


**Supplementary:**
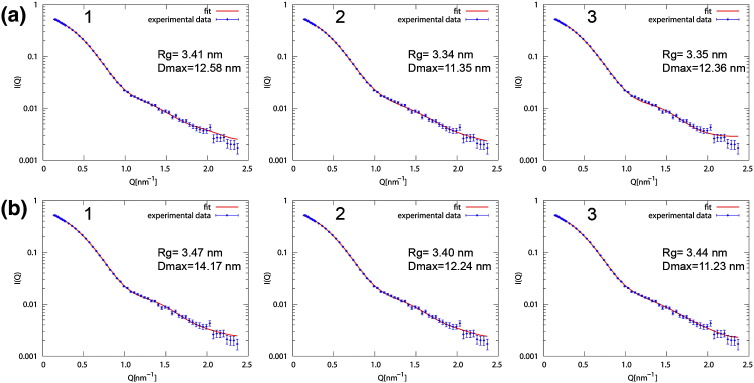


## Footnotes

‡ www.analyticalultracentrifugation.com

§ http://www.ill.fr/lss/grasp/grasp_main.html

║ www.embl-hamburg.de/ExternalInfo/Research/Sax/software.html

∥ https://genesilico.pl/toolkit/unimod?method=MetaMQAPII

**a** http://pymol.sourceforge.net/
