# Corrigendum to “HsdR Subunit of the Type I Restriction-Modification Enzyme EcoR124I: Biophysical Characterisation and Structural Modelling” [J. Mol. Biol. 376(2) 2008 Feb 15: 438–452.]^☆^

**DOI:** 10.1016/j.jmb.2020.02.027

**Published:** 2020-03-27

**Authors:** Agnieszka Obarska-Kosinska, James E N Taylor, Philip Callow, Jerzy Orlowski, Janusz M. Bujnicki

**Affiliations:** 1 Laboratory of Bioinformatics and Protein Engineering, International Institute of Molecular and Cell Biology, Trojdena 4, 02-109 Warsaw, Poland; 2 Biophysics Laboratories, Institute of Biomedical and Biomolecular Sciences, University of Portsmouth, PO1 2DT, UK; 3 EPSAM and ISTM Research Institutes, Keele University, Staffordshire ST5 5BG, UK; 4 ILL-EMBL Deuteration Laboratory, Partnership for Structural Biology, Institut Laue Langevin, 38042 Grenoble Cedex 9, Grenoble, France

The concentration of salt in buffer A has been incorrectly documented in Materials and Methods, and the Results section. It should read as “buffer A (10 mM Tris–HCl (pH 8.0), 50 mM NaCl, 1 mM Na_2_EDTA)” and not “buffer A (10 mM Tris–HCl (pH 8.0), 100 mM NaCl, 1 mM Na_2_EDTA)”.

The authors would like to apologize for any inconvenience caused.

